# Effect Modification of the Association between Short-term Meteorological Factors and Mortality by Urban Heat Islands in Hong Kong

**DOI:** 10.1371/journal.pone.0038551

**Published:** 2012-06-22

**Authors:** William B. Goggins, Emily Y. Y. Chan, Edward Ng, Chao Ren, Liang Chen

**Affiliations:** 1 Division of Biostatistics, School of Public Health and Primary Care, Chinese University of Hong Kong, Shatin, Hong Kong; 2 Division of Family Medicine, School of Public Health and Primary Care, Chinese University of Hong Kong, Shatin, Hong Kong; 3 Department of Architecture, Chinese University of Hong Kong, Shatin, Hong Kong; National University of Singapore, Singapore

## Abstract

**Background:**

Prior studies from around the world have indicated that very high temperatures tend to increase summertime mortality. However possible effect modification by urban micro heat islands has only been examined by a few studies in North America and Europe. This study examined whether daily mortality in micro heat island areas of Hong Kong was more sensitive to short term changes in meteorological conditions than in other areas.

**Method:**

An urban heat island index (UHII) was calculated for each of Hong Kong’s 248 geographical tertiary planning units (TPU). Daily counts of all natural deaths among Hong Kong residents were stratified according to whether the place of residence of the decedent was in a TPU with high (above the median) or low UHII. Poisson Generalized Additive Models (GAMs) were used to estimate the association between meteorological variables and mortality while adjusting for trend, seasonality, pollutants and flu epidemics. Analyses were restricted to the hot season (June-September).

**Results:**

Mean temperatures (lags 0–4) above 29°C and low mean wind speeds (lags 0–4) were significantly associated with higher daily mortality and these associations were stronger in areas with high UHII. A 1°C rise above 29°C was associated with a 4.1% (95% confidence interval (CI): 0.7%, 7.6%) increase in natural mortality in areas with high UHII but only a 0.7% (95% CI: −2.4%, 3.9%) increase in low UHII areas. Lower mean wind speeds (5^th^ percentile vs. 95^th^ percentile) were associated with a 5.7% (95% CI: 2.7, 8.9) mortality increase in high UHII areas vs. a −0.3% (95% CI: −3.2%, 2.6%) change in low UHII areas.

**Conclusion:**

The results suggest that urban micro heat islands exacerbate the negative health consequences of high temperatures and low wind speeds. Urban planning measures designed to mitigate heat island effects may lessen the health effects of unfavorable summertime meteorological conditions.

## Introduction

Studies on the short term association between weather conditions and mortality have generally found that human mortality tends to increase during periods of cold or hot weather with a U- J- or reversed J- shaped association being evident [1]–[6].

While it has been well-established that mortality tends to rise in urban areas when the temperature exceeds a certain threshold, which varies from city to city, few studies have looked at variation in this effect within cities. An important potential modifier of these heat effects is the presence of the urban heat island (UHI) effect due to the thermal capacity of buildings and sealing surfaces with artificial materials, which can cause temperatures in parts of cities characterized by large areas covered by buildings, roadways, parking areas, narrow streets and lack of green space, to substantially exceed those of greener and less dense rural and suburban areas [7]. Only a few studies have examined this potential effect modifier and none have looked at meteorological variables other than temperature.

A study conducted in Montreal looked at the association between mortality and high temperatures in the summertime and conducted stratified analyses where the strata were defined according to surface temperatures recorded from two thermal surface images [7]. This study found that the elevated risk of death on hot days was significantly greater in heat island areas, defined as areas of the city with higher thermal surface temperatures. A study of heat wave related mortality in Berlin, Germany, found that mortality increases during heat waves were greater in districts with a higher proportion of land area covered by sealed surfaces [8], while a case-control study found that higher surface temperatures around residences estimated from satellite images were a significant risk factor for mortality during the 2003 heat wave in France [9].

Hong Kong is a Special Administrative Region (SAR) of China with a total land area of 1104 km^2^ and a 2010 population of 7,061,200. The SAR consists of a densely populated central urban area (occupying around 25% of the land area), suburban ‘New Towns’ (occupying around 30% of the land area), which are also sometimes quite densely populated, and villages and rural areas (occupying around 45% of the land area). The overall population density is 6480 per km^2^, among the highest in the world, but density in urban areas is far higher than this [10]. For instance the most densely populated district, Kwun Tong, has a population density of 53110 per square km [10]. Hong Kong has a sub-tropical climate with hot and humid summers. The summer average temperature is around 28 degree C and the average relative humidity is around 75%. It has had one of the world’s highest average increases in urban ambient temperature during the past century [11].

As previous studies of micro heat island effects on mortality increases during hot weather have all been done in Europe or North America, cities with cooler climates and lower population densities, little is known about the this effect in the generally more densely populated and hotter cities in Asia. In this study we examine the way in which the association between hot weather and mortality varies between areas of Hong Kong with different degrees of urban heat island index (UHII) for the period 2001–2009.

## Materials And Methods

### Ethics Approval

Ethical approval for the study was obtained from the Joint Chinese University of Hong Kong-New Territories East Cluster Clinical Research Ethics Committee.

### Data

Microdata sets containing data on all deaths in Hong Kong from 2001–2009 were obtained from the Hong Kong Census and Statistics Department. Variables available in the data include date of death, cause of death (ICD-10), gender, age and tertiary planning unit (TPU) of residence for each decedent. Data on meteorological variables including mean daily temperature, mean relative humidity (RH), total daily global solar radiation (GSR) and mean daily wind speed were obtained from the Hong Kong Observatory. Mean temperature and relative humidity were measured at the centrally located Hong Kong Observatory headquarters. Total GSR was measured at King’s park, which is also centrally located, while mean wind speed was measured at Waglan Island, which is away from the city. Data on average daily pollutants including particulate matter <10 µm (PM_10_), nitrogen dioxide (NO_2_), sulfur dioxide (SO_2_), and ozone (O_3_) for 14 monitoring stations were obtained from the Hong Kong Environmental Protection Department. The levels for each day were then computed as the average across the 14 stations. Data on influenza consultation rates per 1000 consultations for general practitioners in Hong Kong were obtained from the Hong Kong Department of Health website.

Physiological Equivalent Temperature (PET) has been used to assess urban human thermal comfort [12]. The model takes into account environmental factors like air temperature, air humidity, wind velocity and mean radiant temperature to assess the thermal comfort levels of an individual. Based on user surveys, it is possible to establish the neutral PET value of inhabitants of the city. For example, in the hot and humid summer months of Hong Kong, the neutral PET is around 27 to 29°C [13]. To bio-climatically calculate the PET value of an urban area, the near ground level air temperature due the UHII of the locality and wind availability needs to be estimated.

For this study, the UHII for each of the tertiary planning units in Hong Kong was calculated using land use and building geometry data provided by the Planning Department of the Hong Kong SAR Government. Using an ArcGIS embedded macro, the point and continuous sky view factor (SVF) values for the city were calculated, and an SVF map was generated. By correlating urban morphological data with the SVF at an area average grid cell of 100 m×100 m, the UHIIs can be correlated with ground level measurements using mobile stations in selected areas in urban Hong Kong, and predicted [14]. The regression analysis indicated that SVF values have a close negative relationship with UHII [14]. Statistical studies also indicated that UHII is better correlated with SVF values based on 100 m radius neighbourhood average [14].

The greenery coverage of the urban area was estimated using a satellite image. The 100 m×100 m grid cell normalized difference vegetation index (NVDI) was computed. Greenery’s contribution to lowering UHII was factored based on the area average greening coverage of the urban areas [15]. Using the Envimet model simulation software, which had been calibrated based on field studies in Hong Kong, the cooling effects of vegetation can be predicted. In general, 30% tree/shrub coverage may lower by 1 degree C the urban air temperature on a typical hot summer day in Hong Kong.

The area average wind velocity was assessed based on the frontal area densities (FAD) of the urban forms [16]. Urban morphological data has been used to calculate the 100 m×100 m area average FAD of different urban areas. This was then compared with experimental wind tunnel results of 20 selected urban areas in Hong Kong. The regression analysis indicates that FAD values have a close positive relationship with lower urban air ventilation availability.

Based on a field survey using globe-temperature micro-stations in urban areas of Hong Kong, the mean radiant temperature (Tmrt) was calculated and estimated to be 2 to 4 °C above air temperature based on the human body standing under shades provided by the surrounding buildings [17].

By feeding the various environmental parameters into the PET model, a bio-climatic map of the urban areas of Hong Kong was drawn. The bioclimatic map indicates the intra-urban PET differences of the urban areas. The difference is due purely to buildings and the physical and artificial urban development. Each 100 m×100 m area was classified into 8 ordinal classes. In general the PET will be 1°C higher for each one unit increase in class. Thus class 8 areas, which are the densest urban areas, can have a PET value 7°C higher than class 1 areas. Class 3 is considered to be neutral, representing ambient conditions at sea level in summer in Hong Kong in the absence of heat island effects. Class 1 and 2 areas experience lower PET than ‘neutral’ areas due to their higher elevation. The map gives a good indication of the thermal environment inhabitants may experience. Given the same ambient thermal environment, those living in class 8 areas will experience the highest level of heat stress, those in class 1 areas the lowest, and classes in between will experience intermediate levels of heat stress. The mean class for the TPU was then the average of the classes for all inhabited 100 m×100 m grid areas in the TPU.

### Statistical Methods

Poisson Generalized Additive Models (GAMs) [18] were used to model the association between meteorological variables and mortality while controlling for important confounders. Analyses were restricted to the hot season, June – September, and included the years from 2001 to 2009. Models were fit for all daily natural deaths and daily non-cancer natural deaths as non-cancer mortality was found to be more sensitive to heat effects in our previous study [11]. Natural and non-cancer deaths were stratified according to the UHII for the tertiary planning unit of residence of the decedent. Specifically TPUs with mean UHII greater than the overall median of 3.67 were classified as ‘hot’ areas and those below the median as ‘cool’ areas. Note that this ‘median’ value was calculated such that about 50% of Hong Kong’s population lives in TPUs with mean UHII >3.67 and 50% in TPUs with UHII <3.67. Separate models were then fit for each of four outcomes: (1) natural deaths in ‘cool’ TPUs, (2) natural deaths in ‘hot’ TPUs, (3) non-cancer deaths in ‘cool’ TPUs, and (4) non-cancer deaths in ‘hot’ TPUs. Other than the stratification of the analysis according to the UHII of each TPU there was no spatial element in the models. Initial models included a day of the year indicator, 1, …, 365 (or 366 for leap years) with maximum degrees of freedom (df) for the GAM spline curve = 4 to control for seasonality, a day of study variable, 1,…, 1098, also with maximum df = 4 to control for long-term trends due to changes in population size, age structure, and medical advances, and a day of the week indicator which was treated as a categorical variable. Meteorological variables included in the models included mean temperature, mean relative humidity, daily total solar radiation, and the natural logarithm of mean wind speed (log transformed to reduce the influence of outliers). After examination of the time course of associations using distributed lag models it was decided to use the mean of the same day and previous 4 days (lags 0–4) for each of these variables in subsequent models. Each meteorological variable was initially allowed a maximum of 4 df. After graphical examination of the associations it was decided to use linear terms for log wind speed and solar radiation, and two hockey stick terms for mean temperature, one term T_HOT_  = T –29 if mean daily temperature >29°C and 0 otherwise, and T_COLD_  = 28– T if mean temperature <28°C and 0 otherwise. The thresholds of 28°C and 29°C were chosen since a U-shaped association between temperature and mortality was found in the graph with clear increases in mortality below 28°C and above 29°C. Relative humidity was modeled using a smooth term as its relationship with mortality was inverse U-shaped and very non-significant thus it was included as a confounder only. Mean daily PM_10_ concentrations were included to control for pollutant levels. The means of same day and previous day’s concentration were used as a previous study [19] found this to be the strongest predictor of mortality and this variable was log_10_ transformed to reduce the influence of outliers. R version 2.9.2 statistical software [20] was used for all analyses. The R package mgcv [18] was used to fit the GAM models, in conjunction with the dlnm [21] package which was used for the distributed lags modeling. The ‘quasipoisson’ option was used for the distribution family so as to allow for the possibility of overdispersion (variance greater than the mean) of the outcome variable [22]. The partial autocorrelation of the residuals for each of the models were checked to see if autoregressive terms were necessary in the model. The mgcv package uses cross-validation to select the degrees of freedom (df) for nonparametric smooth terms, and thus allows for flexible modeling of non-parametric associations while protecting against over-fitting [18]. To examine the possibility that the results could be confounded by differences in socioeconomic status between residents living in ‘hot’ areas and those living in ‘cool’ areas we further stratified residence by median household income, with those living in TPUs with median household income ≥ HK$17,300 (US$2,220) per month being classified as high SES and those with median household income less than this figure as low SES (this split the total deaths roughly in 2). Stratified analyses were then conducted for 4 strata: high UHI and low SES, low UHI and low SES, high UHI and high SES and low UHI and high SES.

## Results

**Figure 1:**
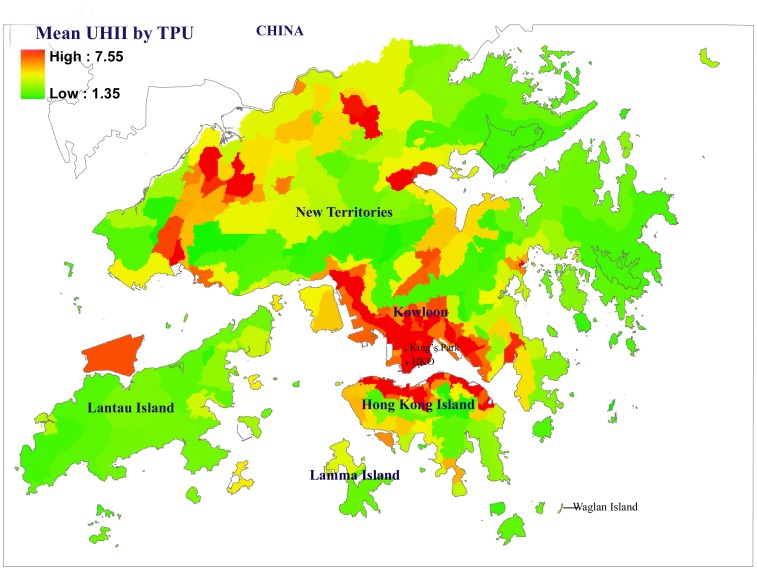
Chloropleth map showing mean Urban Heat Island Index (UHII) for Tertiary Planning Units (TPUs) in Hong Kong.

Descriptive statistics for the study variables are shown in table 1. Hong Kong generally has relatively low variation in mean temperatures and humidity during the summer months. Figure 1 displays a map showing the geographic distribution of UHII by TPU in Hong Kong, along with the locations of the stations which provided each meteorological measurement.

**Table 1:** Descriptive statistics for study variables, Hong Kong, June-September, 2001–2009.

Variable	Mean (SD)	Median	25–75%ile	5–95%ile	Min-Max
Mean Temp°C	28.3 (1.5)	28.6	27.4–29.5	25.8–30.3	22.2–31.8
Wind Speed km/hr	20.5 (10.6)	18.5	12.6–26.1	7.6–39.9	3.2–70.0
Solar Radiation MJ/m^2^	15.9 (6.8)	15.9	9.9–20.7	3.7–25.4	0.9–27.9
Relative Humidity (%)	80.8 (7.4)	80	76–86	70–93	51–98
Daily Natural Deaths	80.7 (10.4)	81	74–88	64–98	49–115
Heat Island Areas	39.2 (7.5)	39	34–44	27–50	6–61
Non-Heat Island Areas	38.3 (7.1)	38	34–43	27–43	11–64
Daily Non-Cancer Deaths	49.1 (7.9)	49	44–54	37–62	23–78
Heat Island Areas	23.9 (5.7)	24	20–28	16–34	4–43
Non-Heat Island Areas	22.2 (5.2)	22	19–25	14–31	5–40

### Natural Cause Mortality

Results for models fit for natural cause mortality are shown in table 2. Results of stratified analyses show that the high temperature effect is significant only for the heat island areas, with an average rise in mortality of 4.1% for a 1°C rise in lag 0–4 temperature above 29°C (p = .019). The high temperature effect in the non heat island areas is lower, 0.7% rise for 1 C increase above 29°C, and non-significant (p = .66). However the difference in coefficients is not statistically significant (p = .16). The cool temperature effect below 28°C was non-significant in the all Hong Kong analysis, and in both of the stratified analyses. The biggest difference between heat island and non-heat island areas is in the association of wind speed with mortality. For heat island areas the association is highly significant (p = .0002) with very calm recent conditions (5^th^ %ile lag 0–4 mean wind speed, corresponding to11.0 km per hour) being associated with a 5.7% higher average mortality than very windy recent conditions (95%ile lag 0–4 mean wind speed, corresponding to 33.6 km/hour). Note that although we used the natural logarithm of mean wind speed in the models we can express the percentage increase in mortality in terms of mean wind speed since the 5^th^ and 95^th^ percentiles for mean wind speed are just the antilogs of the corresponding percentiles for log mean wind speed. For non-heat island areas the association with wind speed is slight and non-significant (p = .83). Also the difference in wind speed coefficients between hot and cool areas is statistically significant (p = .0052). The solar radiation effect was not significant (p = .48) overall with 1.5% (95% CI = −2.5, 5.6) higher mortality when lag 0–4 solar radiation was at the 95^th^ % ile vs. the 5^th^ %ile. The solar radiation effect was also not significant in the stratified analyses but the magnitude of effect was stronger for cool areas. The partial autocorrelation of the residuals for both models were all <0.1 and non-significant indicating that adding autoregressive terms to the model was not necessary.

**Table 2:** Results of Generalized Additive Models for All natural causes mortality and non-cancer mortality in Hong Kong June-September, 2001–2009*.

	All Areas		Hot Areas		Cool Areas		P-value
All natural-cause	% increase	P- value	% increase	P- value	% increase	P- value	hot vs.
mortality	(95% CI)		(95% CI)		(95% CI)		cool areas
Temp>29°C	2.1 (−0.3, 4.6)	.080	4.1 (0.7, 7.6)	.019	0.7 (−2.4, 3.9)	.66	.16
(per 1°C increase)							
Temp<28°C	0.9 (−0.6, 2.5)	.24	0.6 (−1.2,2.8)	.63	0.8 (−1.1, 2.8)	.43	.88
(per 1°C decrease)							
Mean Wind Speed	2.3 (0.2, 4.5)	.030	5.7 (2.7, 8.9)	.0002	−0.3 (−3.2, 2.6)	.83	.0052
(5^th^ vs. 95^th^ %ile)							
Solar Radiation	1.5 (−2.5,5.6)	.48	−0.6 (−6.4,5.5)	.84	3.1 (−2.7,9.3)	.29	.52
(95^th^ vs, 5^th^ %ile)							
**Natural non-cancer**							
**Mortality**							
Temp>29°C	2.3 (−0.7, 5.3)	.13	5.2 (0.9, 9.8)	.018	2.3 (−1.7, 6.6)	.26	.35
(per 1°C increase)							
Temp<28°C	2.4 (0.5, 4.3)	.012	1.4 (−1.3, 4.0)	.31	0.8 (−1.7, 3.4)	.52	.77
(per 1°C decrease)							
Mean Wind Speed	2.3 (−0.3, 5.1)	.083	4.7 (0.9, 7.6)	.019	-1.6 (−5.3, 2.2)	.40	.024
(5^th^ vs. 95^th^ %ile)							
Solar Radiation	2.0 (−3.0,7.2)	.47	1.9 (−5.4,9.9)	.60	0.5 (−7.8,8.3)	.90	.62
(95^th^ vs, 5^th^ %ile)							

* Adjusted for humidity, RSP, season, trend and day of the week.

† Temp refers to daily mean temperature.

There was a slight tendency for TPUs classified as ‘hot’ based on UHII to also have lower SES. Of the deaths in ‘hot’ TPUs, 54.3% were in low SES TPUs whereas only 45.6% of deaths in ‘cool’ TPUs were also in low SES TPUs. The results of analyses stratified by UHII and SES indicated that both high UHII and low SES appeared to exacerbate the adverse high temperature and low wind speed effects (Table 3). For temperature the estimated mortality increases per 1°C increase above 29°C were 5.6% for high UHII/low SES, 2.6% for low UHII/low SES, 3.0% for high UHII/high SES and -1.2% for low UHII/high SES. For wind speed the estimated mortality increases for lag 0–4 mean wind speed  = 11.0 km/hr (5^th^ %ile) vs. 33.6 km/hr (95^th^%ile) were 8.0% for high UHII/low SES, 3.3% for low UHII/low SES, 2.6% for high UHII/high SES and −3.2% for low UHII/high SES.

**Table 3:** Results of Generalized Additive Models for all natural causes mortality in Hong Kong June-September, 2001–2009 stratified by SES and UHII of TPU of residence.

	Temp>29°C.		Mean Wind Speed	
	(per 1°C. increase)	P-value	(5^th^ %ile vs. 95^th^ %ile)	P-value
	% Increase (95% CI)		% Increase (95% CI)	
Hot/Low SES	5.6 (0.5, 10.9)	.028	8.0 (3.6, 12.7)	.0003
Cool/Low SES	2.6 (−1.9, 7.3)	.26	3.3 (−1.0, 7.7)	.13
Hot/High SES	3.0 (−1.7, 8.0)	.22	2.6 (−1.6, 7.1)	.23
Cool/High SES	−1.2 (−5.2, 3.1)	.59	−3.2 (−7.4, 0.8)	.12

### Non-cancer Mortality

Table 2 also shows the results of the models applied to daily non-cancer natural mortality. As expected, non-cancer mortality showed greater sensitivity to temperature than all natural mortality. The high temperature effect was statistically significant for heat island (p = .018) but not for non-heat island (p = .26) areas and the estimated effect was stronger for heat island (5.2% higher per 1°C rise above 29°C) than for non-heat island (2.3% higher per 1C rise above 29°C) areas. However the difference in coefficients between the two groups was not significant (p = .35). As was the case for all natural mortality the biggest difference between heat island and non-heat island areas for non-cancer mortality was for wind speed, with calm conditions again strongly associated with higher mortality in heat island areas (p = .019) but not in non-heat island areas (p = .40). The difference between coefficients was statistically significant (p = .024). Solar radiation was not significant in any of the analyses and was slightly stronger for heat island areas, but the difference was not significant. The partial autocorrelation of the residuals for both models were all <0.1 and non-significant indicating that adding autoregressive terms to the model was not necessary. Cool temperature effects were also slightly stronger in heat island areas, but this difference was not significant.

## Discussion

Our analyses showed that in Hong Kong, there was a large and significant difference between heat island and non-heat island areas in the effects of wind speed, with recent calm conditions being associated with significantly higher natural and non-cancer mortality in heat island areas but almost no effect in non-heat island areas. This finding is not surprising as studies have found that the urban heat island effect is more intense when the wind speeds are lower [23], [24]. High temperature effects were also considerably stronger for heat island areas, particularly for all natural mortality models, although the differences in temperature effects between heat island and non-heat island areas were non-significant. We did not find relative humidity or solar radiation to be significantly associated with mortality in any of the models. Analysis of the effect of colder temperatures during the cool season in Hong Kong (November-March) found that this effect was virtually identical for the UHI and non-UHI areas (data not shown). While the range of mean temperatures above 29°C in Hong Kong is quite narrow (maximum lag 0–4 average  = 30.9°C) about one-third of days during June-September have mean temperatures above this threshold.

Previous studies [7]–[9] which have looked at effect modification of meteorological effects on mortality by urban characteristics have generally used temperature as the only meteorological variable and have used different methods for defining heat island areas. A study from Montreal [7] defined heat islands as areas of the city which were above the 75^th^ %ile in terms of surface temperatures recorded from two thermal surface images captured by a satellite, found that on days with a mean temperature of 26°C the odds of mortality was 28% higher than on days with a 20°C mean temperature in “hot” areas but only 13% higher for “cool” areas, with non-overlapping confidence intervals. A case-control study of elderly deaths during the French 2003 heat wave found that a 1°C higher temperature index within 200 meters of the home (estimated using Satellite images) was associated with an odds ratio for death of 1.82 [9]. A study from Germany [8] found a positive correlation between district mortality rates and the proportion of each district covered by sealed surfaces during time periods of high heat stress.

While to the author’s knowledge no studies in sub-tropical or tropical areas have looked specifically at micro heat islands, some studies have examined heat island effects by comparing excess mortality due to high temperature between urban areas and suburban/rural areas. A recent study of the heat island effect on mortality during heat waves in Shanghai found that estimated excess mortality due to the 1998 heat wave was 27.3 per 100,000 in urban areas but only 7.0 per 100,000 in exurban areas [25]. A study from Bangladesh estimated a 7.5% rise in all-cause mortality per 1°C increase in lags 0–6 mean temperature above the threshold of 28.8C in urban areas vs. a 1.5% rise for rural areas (with a lower threshold of 28.1°C) [26]. There are limitations to studying heat island effects through examining urban vs. suburban/rural difference in heat-mortality associations. This method would be prone to misclassification as some suburban areas may in fact suffer from heat island effects while some suburban areas may not. Our study found that some areas of Hong Kong’s suburban ‘New Towns’, which often consist of densely populated housing estates with high-rise apartment buildings had higher UHI than some of the urban areas. Similar conditions may also exist in other densely populated Asian cities. Another issue is that urban, suburban and rural populations generally differ from each other in socioeconomic, demographic and other characteristics which modify the high temperature-mortality association.

A major limitation of our study, which is shared by other studies of this nature, is the fact that our meteorological variables were measured outdoors at fixed monitoring stations, and thus may not represent the actual conditions to which individuals are exposed.

In conclusion our study found that the association between daily mortality and two meteorological variables: mean daily wind speed and mean daily temperature, was stronger in areas with higher UHII. This suggests that better urban planning designed to reduce the urban heat island effect may help to mitigate the negative health consequences of high temperatures. The current magnitude of excess heat related mortality in Hong Kong is relatively small, due to the fact that the daily mean temperature is seldom more than 2°C. higher than the threshold temperature of 29°C. However, the sharp increase in mortality, particularly non-cancer mortality, when mean daily temperatures rise above 29°C., suggests that heat-related mortality is likely to be a considerable problem in the future since global warming is expected to greatly increase the number of days with temperatures exceeding this threshold.
